# Case series suggesting an association between sertraline and urinary side effects in a Sheffield child and adolescent mental health services (CAMHS) population

**DOI:** 10.1192/bjo.2021.646

**Published:** 2021-06-18

**Authors:** Sidra Chaudhry, Nisha Alex

**Affiliations:** 1 Sheffield Health and Social Care NHS Foundation Trust; 2 Sheffield CAMHS

## Abstract

**Aims:**

To suggest a link between sertraline and urinary side effects in a Sheffield Child and Adolescent Mental Health Service population.

**Background:**

Evidence suggests that Serotonin has an important role in bladder control through central and peripheral neurological pathways. Increased serotonergic activity leads to parasympathetic inhibition, which results in urine retention. It is through this mechanism of action and their effect on pre-synaptic serotonin 1A and peripheral 5-HT3 receptors that SSRIs were observed to have anti-enuretic effect. At low 5-HT concentrations, micturition is inhibited whereas at high levels, an excitatory effect is achieved. This may suggest a dose-dependent relationship between Sertraline and urinary side effects.

**Method:**

Inclusion criteria:

Under 18 years of age

On Sertraline

Reported urinary side effects

Exclusion criteria:

Above 18 years

Not on Sertraline

Associated urinary problems

Did not report urinary side effects

Clinical records of eligible patients were accessed to gauge temporal relationship between initiation of sertraline and reported urinary side effects.

**Result:**

Three cases were identified in the authors’ clinical practice at Sheffield CAMHS that were suggestive of a link between sertraline and urinary side effects.

**Conclusion:**

It's important for clinicians to bear in mind the genitourinary side effects of SSRIs, which may be debilitating for patients in the CAMHS population. It's equally important for us as clinicians to educate young people and their parents about these potential side effects and how they can be managed. It has also been observed that higher doses of Sertraline have shown a possible link between onset of urinary side effects.

